# Phase I Trial of a *Lactobacillus crispatus* Vaginal Suppository for Prevention of Recurrent Urinary Tract Infection in Women

**DOI:** 10.1155/2007/35387

**Published:** 2007-11-26

**Authors:** Christopher A. Czaja, Ann E. Stapleton, Yuliya Yarova-Yarovaya, Walter E. Stamm

**Affiliations:** Division of Allergy and Infectious Diseases, University of Washington School of Medicine, Seattle, WA 98195, USA

## Abstract

*Objectives*: We performed a phase I trial to assess the safety and tolerance of a *Lactobacillus* vaginal suppository for prevention of recurrent UTI. *Methods*: Premenopausal women with a history of recurrent UTI were randomized to use *L. crispatus* CTV-05 or placebo vaginal suppositories daily for five days. *Results*: 30 women were randomized (15 to *L. crispatus* CTV-05). No severe adverse events occurred. Mild to moderate vaginal discharge and genital irritation were reported by women in both study arms. Seven women randomized to *L. crispatus* CTV-05 developed pyuria without associated symptoms. Most women had high concentrations of vaginal ${\text{H}}_{2}0_{2}$-producing lactobacilli before randomization. *L. crispatus*, *L. jensenii*, and *L. gasseri* were the most common *Lactobacillus* species identified, with stable prevalence over time. *Conclusions: L. crispatus* CTV-05 can be given as a vaginal suppository with minimal sideeffects to healthy women with a history of recurrent UTI. Mild inflammation of the urinary tract was noted in some women.

## 1. INTRODUCTION

Urinary tract infections (UTIs) affect millions of women each year, with an annual societal cost of billions of dollars [1]. Importantly, more than one quarter of women with a UTI will have a recurrent infection within six months [2]. There are few established options for prevention of UTI other than the use of prophylactic antibiotics [3]. However, resistance to commonly used antibiotics is increasing among bacterial cystitis isolates [4]. Therefore, effective nonantibiotic methods of prevention are needed. One potential alternative may be a lactobacillus probiotic.

A growing body of evidence suggests that vaginal H_2_0_2_-producing lactobacilli may have a protective effect against urogenital infections, including UTI [5–11]. It is hypothesized that lactobacilli prevent uropathogen colonization of the vagina, a necessary step in ascending infection of the bladder. Several clinical trials have demonstrated that certain *Lactobacillus* species, mainly *L. rhamnosus* and * L. fermentum*, can be given orally or vaginally with resulting colonization of the vagina, reduction in vaginal coliform counts, and even reduction in UTI recurrence [12–18]. Specific strains were chosen for their abilities to produce H_2_0_2_, adhere to uroepithelial cells, interfere with uropathogen attachment and growth, and persist in the vagina [18, 19]. The most frequently existing vaginal *Lactobacillus* species, *L. crispatus* [20, 21], also produces H_2_0_2_ and possesses the adherence and inhibitory qualities essential for a good probiotic candidate [22, 23].

*L. crispatus* strain CTV-05 has been developed as a vaginal suppository (LACTIN-V) for the prevention of recurrent UTI in women. *L. crispatus* CTV-05 is highly adherent to vaginal epithelial cells [24], and when given as a suppository can colonize the vagina [25]. Furthermore, *L. crispatus* CTV-05 has been reported to have a specific DNA fingerprint that distinguishes it from endogenous vaginal lactobacilli [25]. To further evaluate the safety of this new formulation of *L. crispatus* CTV-05 and its effect on the vaginal microbial flora, we conducted a phase I, randomized, double-blind, placebo-controlled trial of vaginal *L. crispatus* CTV-05 in premenopausal women with a history of recurrent UTI.

## 2. SUBJECTS AND METHODS

**Participants**

We recruited premenopausal women aged 18–35 years with a history of three or more uncomplicated UTIs diagnosed in the past year, or two uncomplicated UTIs diagnosed in the past six months, from (student health center, University of Washington, Wash, USA.) Additional eligibility requirements included regular menstrual cycles or amenorrhea for at least six months secondary to use of a hormonal contraceptive, a normal Pap smear documented in the last year or at the baseline clinic visit, abstinence from sexual activity or participation in a mutually monogamous sexual relationship, use of birth control, agreement not to use other intravaginal products, agreement not to have sexual intercourse or use tampons between the baseline and first follow-up visit, and capability to understand English and provide informed consent. Exclusion criteria included history of urologic abnormality, recent urologic surgery or urinary catheterization, history of complicated pyelonephritis or renal calculi, hysterectomy, recent sexually transmitted infection (STI) or bacterial vaginosis, risk factors for STI and HIV, history of recurrent genital herpes, menses anticipated within ten days, pregnancy, lactation, recent antibiotic or antifungal use, diabetes or other immunocompromised state, drug or alcohol abuse, use of the (NuvaRing), prior use of the study drug or allergy to any of its components, and abnormal initial pelvic examination. The study was approved by (Institutional Review Board (IRB), University of Washington, Wash, USA.)

**Study design**

Subjects were randomized in a double-blinded fashion to *L. crispatus* CTV-05 at a dose of 5 × 10^8^ colony forming units (cfu) or placebo vaginal suppository to be inserted daily for five days. *L. crispatus* CTV-05 and placebo suppositories were similar in appearance and consisted of a preservation matrix and maltodextran. Both were prepackaged by the manufacturer (Osel, Inc., Palo Alto, Calif, USA) according to a randomization schedule and supplied to the study site sequentially labeled with a subject number.

Subjects were seen at three clinic visits over a period of one month. At the first (baseline) visit, eligible subjects provided informed consent before undergoing a structured medical and gynecologic history and physical examination. They provided urine, vaginal, and cervical specimens. Subjects inserted the first dose of study drug in the clinic and were instructed to record any symptoms occurring during the first week of study on a prepared diary card. Subjects were seen in follow-up 6–8 days (1-week) visit and 26–34 days (4-week) visit after enrollment. During these visits, new symptoms, new urogenital infections, and any changes in clinical history were recorded. Follow-up visits were otherwise similar to the baseline visit. Finally, subjects were contacted by telephone six months after enrollment to assess symptoms, pregnancy, new diagnoses, or major medical events.

Laboratory testing included urine dipstick testing, urinalysis, urine culture, vaginal fluid wet preparation slides, gram stains, and culture for facultative isolates and *Lactobacillus* species at all visits. Repetitive element sequence-based polymerase chain reaction (rep-PCR) for *L. crispatus* CTV-05 was performed on three lactobacillus colonies from culture at each visit [25]. Urine pregnancy testing was performed at the baseline and 4-week visits. Testing for *Neisseria gonorrhoeae* and *Chlamydia trachomatis* by DNA amplification was done at the baseline visit.

A sample size of 30 was chosen to evaluate the primary outcome of safety as assessed through self-reported symptoms, physical exam findings, and laboratory studies. Secondary outcomes included shifts in the vaginal flora assessed by vaginal culture and vaginal colonization with *L. crispatus* CTV-05 assessed by rep-PCR. The Fisher exact test was used to test statistical significance.

## 3. RESULTS

**Figure 1:**
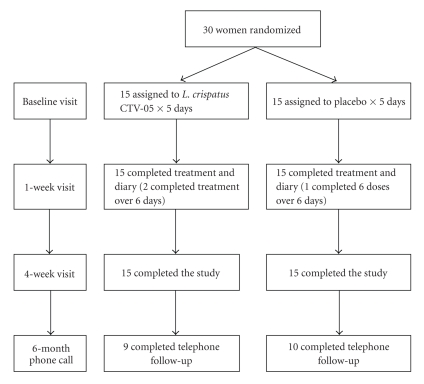
Randomization and followup of subjects.

Thirty women were randomized (15 to *L. crispatus* CTV-05). All subjects took the five planned doses of study drug. Two women randomized to *L. crispatus* CTV-05 completed treatment over six days, and one woman randomized to placebo used six suppositories over six days. All women remained on study through the 4-week visit (Figure 1).

Baseline characteristics of subjects were similar in the two treatment groups (Table 1). The majority of women were young, white, healthy, university students in their 20s. The median number of UTIs in the last year was three. Screening tests for pregnancy and STI were negative for all subjects.

**Safety**

There were no severe adverse events. Mild to moderate adverse events were relatively common, however. Of those felt by the investigators to be related to study drug use, abnormal vaginal discharge was the most frequently occurring, followed by external genital irritation and vaginal candidiasis (Table 2). These adverse events occurred with similar or greater frequency in women randomized to placebo as compared to women randomized to *L. crispatus* CTV-05. Three women reporting vaginal candidiasis also reported multiple prior episodes in the preceding 12 months. Two women randomized to *L. crispatus* CTV-05 reported episodes of cystitis during the study.

All subjects completed a diary card of symptoms that occurred during the period of study drug insertion. Recorded symptoms generally confirmed the reported adverse events and were similar to symptoms reported at the 1-week clinic visit (below). These symptoms occurred with similar frequency in each treatment arm.

**Figure 2:**
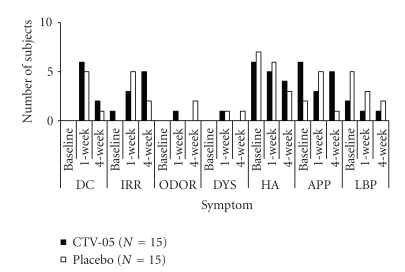
Symptoms reported by ≥2 women at clinic visits (*P>.05* for comparison of *L. crispatus* CTV-05 to placebo at all visits). CTV-05, *L. crispatus* CTV-05; DC, abnormal vaginal discharge; IRR, external genital or vaginal irritation; ODOR, vaginal odor; DYS, dysuria; HA, headache; APP, abdominal or pelvic pain/cramps; LBP, low back pain.

Abnormal vaginal discharge was the symptom most frequently reported by subjects at clinic visits following study drug use (Figure 2). While no women reported abnormal vaginal discharge at baseline, six randomized to *L. crispatus* CTV-05 and five randomized to placebo reported abnormal vaginal discharge at the 1-week visit. Abnormal discharge was less frequently reported at the 4-week visit. External genital or vaginal irritation was also reported by six women in each treatment arm at follow-up visits. Vaginal odor was reported at the 1-week visit by one woman randomized to *L. crispatus* CTV-05, and at the 4-week visit by two women randomized to placebo. Dysuria was reported by one woman randomized to *L. crispatus* CTV-05 at the 1-week visit and by one woman randomized to placebo at the 1- and 4-week visits. Headache, abdominal or pelvic cramps/abdominal pain, and low back pain were frequently reported at baseline as well as at follow-up visits. There were no significant differences in reported symptoms at any visit between women in the two treatment arms.

Physical exam findings were benign in both treatment arms. External genital erythema was found at the 1- and 4-week visits in more women randomized to *L. crispatus* CTV-05 than in women randomized to placebo (4 versus 1, P=.33). Moderate-to-profuse vaginal discharge was frequently found in women from both treatment arms during follow-up examinations (12 versus 8, *P* = 1.00), though this finding was also often apparent at the baseline visit (6 women in each arm). One woman randomized to placebo had vaginal erythema noted at 1 week. No cervical, urethral, uterine, or adnexal abnormalities were noted.

Seven (47%) women randomized to *L. crispatus* CTV-05 had nine episodes of pyuria with a urine white blood cell count ≥8 per mm^3^ [26] (range 8–203) during a follow-up clinic visit (Table 3). None of these women had pyuria at baseline. In contrast, none of the women randomized to placebo had pyuria at 1 or 4 weeks (*P* = .01). Pyuria was positively correlated with urine leukocyte-esterase test results (Spearman *r* = 0.57, P<.01). There were no significant associations with vaginal or urinary symptoms or exam findings. Microbes isolated from the urine of women in the *L. crispatus* CTV-05 arm with pyuria included mixed gram-positive organisms (6), lactobacillus (3), *E. coli* (1), enterococcus (1), group B streptococcus (1), and yeast (1). One subject had a negative urine culture, and none had symptomatic infection. Lactobacillus and mixed gram-positive rods were isolated at 10^3^-10^4^ cfu/ml from more women with pyuria (50% and 75%) than without (19% and 46%, resp.) at the 1-week visit (P=.22 for lactobacillus, P=.60 for mixed gram-positive rods) but not at the 4-week visit. None of the other isolated organisms was found at a significantly increased frequency in women randomized to *L. crispatus* CTV-05 versus placebo or in women with pyuria compared to those without pyuria at any visit. Two women randomized to *L. crispatus* CTV-05 and one randomized to placebo had hematuria at the 4-week visit (≥30 red blood cells per mm^3^).

Nine women in the *L. crispatus* CTV-05 arm and ten in the placebo arm completed the six-month follow-up telephone call. None reported pregnancy, and no major health problems occurred. Four women in the *L. crispatus* CTV-05 arm and one in the placebo arm reported one or more episodes of cystitis.

**Effects on vaginal flora**

28 of 30 women had vaginal colonization with H_2_0_2_-producing lactobacilli at baseline and all follow-up visits (Table 4). Two women randomized to placebo had no vaginal H_2_0_2_-producing lactobacilli detected at any clinic visit. One of these women had large quantities of *Gardnerella* and *Bacteroides* species morphotypes on gram stain. Cultures from women with H_2_0_2_-producing lactobacilli yielded heavy (4+) growth.

Vaginal cultures from five women in each treatment arm yielded *E. coli* from one or more clinic visits. *E. coli* was isolated from vaginal cultures taken at the 1- and 4-week visits from 4 women randomized to *L. crispatus* CTV-05 and from one women randomized to placebo (P=.33). No inverse association between vaginal colonization with H_2_0_2_-producing lactobacilli and vaginal colonization with *E. coli* was detected. Vaginal cultures frequently yielded *Candida* at baseline as well as follow-up visits.

**Rep-PCR**

Using rep-PCR, *L. crispatus* CTV-05 was detected in the vaginas of four women randomized to *L. crispatus* CTV-05 (three women at each clinic visit). Three women had a positive assay for *L. crispatus* CTV-05 detected at the baseline visit before administration of the study drug. No women randomized to placebo had a positive assay at any visit (*P* = .22 each visit).

**Figure 3:**
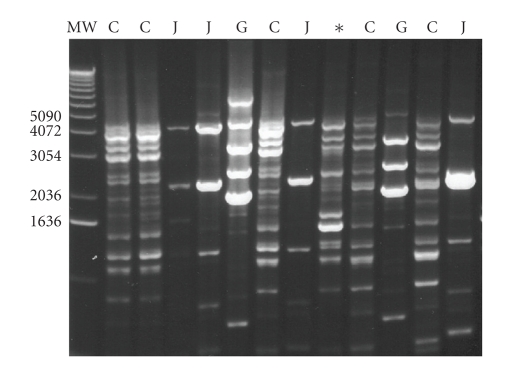
Representative rep-PCR DNA fingerprints from vaginal lactobacillus isolates. MW, Molecular weight standard; C, *L. crispatus*; J, *L. jensenii*, G, *L. gasseri*; *, *L. crispatus* CTV-05.

**Figure 4:**
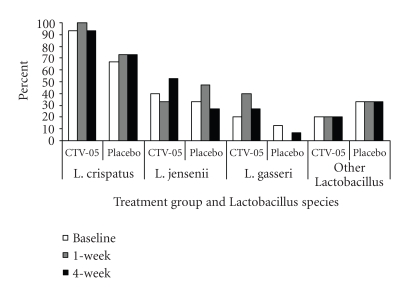
Prevalence of vaginal *Lactobacillus* species at clinic visits (*N* = 15 for each treatment group. P>.05 for comparison of *L. crispatus* CTV-05 to placebo at all visits except for *L. gasseri* at 1-week (P=.02)). CTV-05, *L. crispatus* CTV-05.

We compared our rep-PCR results (Figure 3) to previously published rep-PCR patterns [25] in order to identify the bacterial species. At baseline, 80% of subjects had *L. crispatus*, 37% had *L. jensenii*, 17% had *L. gasseri*, and 27% had other *Lactobacillus* species detected in vaginal culture. The following combinations of *Lactobacillus* species were found: *L. crispatus* (27%), *L. crispatus* and *L. jensenii* (20%), *L. crispatus* and *L. gasseri* (7%), *L. crispatus* and other *Lactobacillus* species (13%), *L. crispatus*, *L. jensenii*, and *L. gasseri* (3%), *L. crispatus*, *L. jensenii*, and other *Lactobacillus* species (7%), *L. crispatus*, *L. gasseri*, and other *Lactobacillus* species (3%), *L. jensenii* (7%), *L. gasseri* (3%), other *Lactobacillus* species (3%), and no *Lactobacillus* (7%). The prevalences of individual species were relatively stable over time (Figure 4). There were no significant differences in *Lactobacillus* species prevalence between subjects in the two treatment arms with the exception of *L. gasseri* at the 1-week visit (*P* = .02).

## 4. DISCUSSION

In phase I, placebo-controlled trial, use of a *L. crispatus* CTV-05 vaginal suppository, for the prevention of recurrent UTI was well tolerated with minimal side effects. There were no serious adverse events, and despite the occurrence of abnormal vaginal discharge and external genital or vaginal irritation in several women, compliance was high. These mild to moderate symptoms appear to be secondary to the act of suppository use or a reaction to the preservation matrix rather than a consequence of *L. crispatus* CTV-05 as they occurred with similar frequency among women in each treatment arm. Several women also reported vaginal candidiasis. However, given the frequency of recurrent vaginal candidiasis experienced by subjects prior to enrollment and the prevalence of *Candida* in baseline vaginal cultures, the relationship of this condition to study drug use is not compelling.

*L. crispatus* CTV-05 use was associated with pyuria (detected by microscopy and urine leukocyte-esterase) in seven women at either the 1- or 4-week visits (*P* = .04 at 4 weeks). Pyuria was not associated with urogenital symptoms, exam findings, or symptomatic UTI. While lactobacillus was not isolated from the urine during every episode of pyuria, it is possible that some lactobacilli were misclassified as mixed gram-positive rods, as both were more frequently found in women with pyuria than in women without. Our data suggest that vaginal instillation of *L. crispatus* CTV-05 induces a mild inflammatory response in the bladder or vaginal mucosa of some subjects without causing prolonged urogenital infection. The importance of this phenomenon with respect to safety or potential efficacy is unclear. It is possible that induction of an asymptomatic inflammatory response by lactobacilli protects against uropathogen colonization of the vagina or infection in the bladder [27].

Limited existing data from prior studies suggest that *L. crispatus* therapy is safe. Women surveyed after participation in a clinical trial of a vaginal capsule formulation of *L. crispatus* CTV-05 for treatment of bacterial vaginosis rarely reported adverse effects, and those reported were largely related to a perceived difference in vaginal discharge. Satisfaction with the vaginal capsule was high [28]. No adverse effects were reported in a pilot study of *L. crispatus* CTV-05 given intravaginally to a small group of healthy women [25]. Nine women with a history of recurrent UTI reported no adverse effects when given *L. crispatus* strain GA198332 as a vaginal suppository every other day for one year [29]. Studies of other probiotic strains of *Lactobacillus* have indicated that they can be given to women safely with minimal or no side effects [12–18]. To our knowledge, pyuria resulting from vaginal instillation of *Lactobacillus* has not been previously reported, and should be evaluated further in future studies.

We did not see an effect of *L. crispatus* CTV-05 use on vaginal *Lactobacillus* growth. However, most study participants had heavy vaginal growth of H_2_O_2_-producing lactobacilli at baseline, making detection of subsequent changes difficult. Data from a pilot study indicated that *L. crispatus* CTV-05 did not displace other endogenous vaginal lactobacilli. Vaginal colonization was most successful in women lacking vaginal H_2_O_2_-producing lactobacilli at the outset, suggesting that a lactobacillus probiotic may be most effective at establishing vaginal colonization in women with abnormal flora, such as those with bacterial vaginosis or recurrent UTI [25].

To our knowledge, our study represents the first attempt to use rep-PCR to identify the genetic fingerprint of the probiotic strain *L. crispatus* CTV-05 in a larger-scale clinical study. Although none of the placebo recipients were colonized with lactobacillus strains having the characteristic rep-PCR pattern of *L. crispatus* CTV-05 at any time, three women randomized to *L. crispatus* CTV-05 had at least one isolate in their vaginal culture at baseline that bore the characteristic fingerprint. Therefore, colonization of the vagina following suppository administration was difficult to demonstrate. As there are no large-scale surveys of the prevalence and distribution of lactobacilli bearing this fingerprint, we do not know whether the rep-PCR technique failed to distinguish *L. crispatus* CTV-05 from other genetically closely related strains of *L. crispatus* naturally colonizing the vagina at baseline, or whether the identical strain was prevalent in our study population. Of note, subjects in our study had a much higher prevalence of vaginal H_2_O_2_-producing lactobacilli in baseline vaginal cultures than found in previous studies [25]. By comparing our rep-PCR results to previous published rep-PCR patterns [25], we characterized lactobacillus isolates at the species level and found *L. crispatus*, *L. jensenii*, and *L. gasseri* to be the most prevalent species, with relatively stable prevalences over time. Our prevalence data are consistent with what has been previously documented [20, 21]. Additional tests are under way to compare quantitative PCR results for *L. crispatus* in women before and after the introduction of active suppositories or placebo.

Our study was not designed nor statistically powered to evaluate the effect of *L. crispatus* CTV-05 on the rate of UTI recurrence. Therefore, it is difficult to interpret the report of cystitis in two women randomized to *L. crispatus* CTV-05. Other small studies have suggested that use of vaginally administered *L. crispatus* may be associated with a lower rate of UTI recurrence [16, 29].

The major strengths of our study include the placebo-controlled, randomized study design, excellent compliance, and extensive and complete followup. Use of a study diary provided finer details of our subjects’ experiences that confirmed adverse events and symptoms reported from memory at clinic visits. Weaknesses that should be mentioned include the inability to accurately document vaginal colonization with the probiotic strain, and the inherent lack of statistical power due to the small sample size typical of phase I studies. We are currently conducting a phase II trial that will have more power to clarify some of the issues raised in this study.

In conclusion, *L. crispatus* CTV-05 is well tolerated when given as a vaginal suppository to healthy women with a history of recurrent UTI. Mild to moderate side effects related to suppository use occur but do not affect compliance. *L. crispatus* CTV-05 may cause mild asymptomatic inflammation of the lower urinary tract. Timing of administration and efficacy in preventing recurrent cystitis should be evaluated further in larger studies.

## Figures and Tables

**Table 1:** Baseline characteristics of study subjects.

	CTV-05 (*N* = 15)	Placebo (*N* = 15)	All (*N* = 30)
Age (years)
Median	23	21	21.5
Range	18–35	19–32	18–35
UTI in the past 12 months (no.)
Median	3	3	3
Range	2–6	2–6	2–6
Race (no.)
White	13	12	25
Asian	2	2	4
Native American	0	1	1
Hispanic ethnicity (no.)	0	1	1
Marital status (no.)
Single	13	11	24
Partner > 4 months	1	2	3
Married	0	1	1
Divorced	1	1	2
Occupation (no.)
Student	11	10	21
Employed full-time	3	5	8
Unemployed	1	0	1
Major medical problem ever (no.)	1	1	2
Pregnant ever (no.)	2	1	3
Abnormal Pap ever (no.)	1	1	2
Antimicrobial use past 30 days (no.)	1	2	3
Sex in the past 30 days (no.)	11	13	24

CTV-05, *L. crispatus* CTV-05.

**Table 2:** Adverse events related to study drug use.

	CTV-05 (*N* = 15)	Placebo (*N* = 15)	*P*
Abnormal vaginal discharge	6	7	1.00
External genital irritation	1	5	.17
Vaginal Candidiasis	4	2	.65
Vaginal odor	1	0	1.00
Abdominal or pelvic cramps/abdominal pain	0	1	1.00
Dysuria	0	1	1.00

CTV-05, *L. crispatus* CTV-05.

**Table 3:** Subjects with pyuria* at follow-up clinic visits.

	CTV-05 (*N* = 15)	Placebo (*N* = 15)	*P*
Baseline	0	2	.48
1-Week	4	0	.10
4-Week	5	0	.04

CTV-05, *L. crispatus* CTV-05.

*Urine white blood cell count ≥8/mm^3^.

**Table 4:** Subjects with vaginal H_2_O_2_-producing *Lactobacillus* detected at follow-up clinic visits.

		CTV-05 (*N* = 15)					Placebo (*N* = 15*)				
SQ Count		0	1+	2+	3+	4+	0	1+	2+	3+	4+
Number of subjects	Baseline	0	0	0	0	15	2	0	2	0	11
	1-Week	0	0	0	0	15	2	0	0	1	12
	4-Week	0	0	0	0	13**	2	0	0	1	12

CTV-05, *L. crispatus* CTV-05; SQ, semiquantitative.

*There were 2 women in the placebo group with no H_2_O_2_-producing lactobacilli detected at any visit. *P* = 0.48 at each visit for comparison of presence or absence of H_2_O_2_-producing lactobacilli in the *L. crispatus* CTV-05 and Placebo groups.

**All 15 subjects had positive vaginal cultures. Semi-quantitative data missing for 2.
